# A novel sampling method to detect airborne influenza and other respiratory viruses in mechanically ventilated patients: a feasibility study

**DOI:** 10.1186/s13613-018-0396-4

**Published:** 2018-04-17

**Authors:** Alicia B. Mitchell, Benjamin Tang, Maryam Shojaei, Lachlan S. Barnes, Marek Nalos, Brian G. Oliver, Anthony S. McLean

**Affiliations:** 1 School of Life Sciences, University of Technology Sydney, Sydney, NSW 2007 Australia; 2 Department of Intensive Care Medicine, Nepean Hospital, Sydney, Australia; 3 Centre for Immunology and Allergy Research, Westmead Institute for Medical Research, Sydney, Australia; 4 Respiratory Virus Infection Research, Marie Bashir Institute for Infectious Diseases and Biosecurity, Sydney, Australia; 5 Nepean Clinical School, Faculty of Medicine, University of Sydney, Sydney, Australia

**Keywords:** Virus, Intensive care unit, Airborne, Influenza, Ventilator

## Abstract

**Background:**

Respiratory viruses circulate constantly in the ambient air. The risk of opportunistic infection from these viruses can be increased in mechanically ventilated patients. The present study evaluates the feasibility of detecting airborne respiratory viruses in mechanically ventilated patients using a novel sample collection method involving ventilator filters.

**Methods:**

We collected inspiratory and expiratory filters from the ventilator circuits of mechanically ventilated patients in an intensive care unit over a 14-month period. To evaluate whether we could detect respiratory viruses collected in these filters, we performed a reverse transcription polymerase chain reaction on the extracted filter membrane with primers specific for rhinovirus, respiratory syncytial virus, influenza virus A and B, parainfluenza virus (type 1, 2 and 3) and human metapneumovirus. For each patient, we also performed a full virology screen (virus particles, antibody titres and virus-induced biomarkers) on respiratory samples (nasopharyngeal swab, tracheal aspirate or bronchoalveolar fluid) and blood samples.

**Results:**

Respiratory viruses were detected in the ventilator filters of nearly half the patients in the study cohort (*n* = 33/70). The most common virus detected was influenza A virus (*n* = 29). There were more viruses detected in the inspiratory filters (*n* = 18) than in the expiratory filters (*n* = 15). A third of the patients with a positive virus detection in the ventilator filters had a hospital laboratory confirmed viral infection. In the remaining cases, the detected viruses were different from viruses already identified in the same patient, suggesting that these additional viruses come from the ambient air or from cross-contamination (staff or visitors). In patients in whom new viruses were detected in the ventilator filters, there was no evidence of clinical signs of an active viral infection. Additionally, the levels of virus-induced biomarker in these patients were not statistically different from those of non-infected patients (*p *= 0.33).

**Conclusions:**

Respiratory viruses were present within the ventilator circuits of patients receiving mechanical ventilation. Although no adverse clinical effect was evident in these patients, further studies are warranted, given the small sample size of the study and the recognition that ventilated patients are potentially susceptible to opportunistic infection from airborne respiratory viruses.

**Electronic supplementary material:**

The online version of this article (10.1186/s13613-018-0396-4) contains supplementary material, which is available to authorized users.

## Background

Influenza and other respiratory viruses spread via three main transmission routes, namely direct contact, respiratory droplets and airborne transmission. The first two routes (direct contact and respiratory droplets) can be reduced by infection control measures (e.g. hand washing and wearing face masks). The third route, airborne transmission, is difficult to prevent since respiratory viruses are ubiquitous in the environment and virus particles constantly circulate in the air [1]. The concentration of airborne viruses is usually low and insufficient to cause disease in humans; however, in those with a compromised immune system (e.g. critically ill patients), the risk of infection increases dramatically [2].

The risk of infection from circulating respiratory viruses is higher in mechanically ventilated patients compared to non-ventilated patients. These patients have an exposed lower airway (the endotracheal tube bypasses the upper airway defence which normally acts as a physical barrier to airborne viruses). In addition, they have multiple risk factors that may further compromise their host defence system, including local trauma (due to intubation and airway manipulation), a weakened local defence (from a loss of mucociliary clearance and cough reflex) and a diminished immune response (e.g. reduced alveolar macrophages in the lungs). Despite this increased infection risk, the air in the ICU is not routinely sampled for the presence of respiratory viruses because no method is currently available for measuring airborne viruses.

The aim of the present study was to evaluate the effectiveness of a novel sampling method that collected inspired/expired air within the ventilator circuit to allow for the measuring of airborne viruses. Detecting airborne viruses in these patients is technically challenging as the concentration of viruses in the inspired/expired air is usually very low; a substantially large volume of air per sample is required for detection. To address this challenge, we applied a novel approach in which we measured viruses trapped in the ventilator filters of mechanically ventilated patients. The ventilator filters have a large volume of inspired/expired air circulating through them each day, thus making them an ideal medium for sampling airborne viruses. Here, we report the findings of a feasibility study using ventilator filters to detect airborne viruses in mechanically ventilated patients admitted to an intensive care unit.

## Methods

### Patient recruitment

We recruited mechanically ventilated patients in an intensive care unit over a 14-month period. Eligible patients included those (1) over 18 years old; (2) suspected of having pneumonia with a viral aetiology (“flu-like” illness in the preceding 7 days); and (3) mechanically ventilated for at least 24 h. Pneumonia was defined as a new lung infiltrate on chest radiography at hospital admission with symptoms and signs of lower respiratory tract infection. “Flu-like” illness was defined as having at least one symptom from two or more symptoms categories. The symptom categories were as follows: (1) fever, (2) constitutional symptoms (e.g. chill, headache, muscle ache) and (3) respiratory symptoms (e.g. cough, sore throat, nasal congestion). Informed consent was obtained from relatives or the legal guardian of the patient. The study was approved by the human ethics committee of our institution.

### Filter collection

**Fig. 1:**
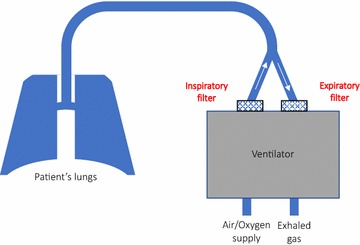
Ventilator circuit with the position of filters shown. A simplified schematic drawing showing the position of the inspiratory and expiratory filters (highlighted in red). Arrows inside each arm of the ventilator circuit indicate the direction of air flow

To evaluate whether we could detect respiratory viruses in the inspired/expired air, we sampled both the inspiratory and expiratory filters from the ventilator circuits (Fig. 1). After the first 24 h of mechanical ventilation, ventilator filters were collected, placed in pre-prepared sample bags and stored in − 80 °C for later processing (see below).

### Processing

Prior to the processing of the filters, care was taken to ensure that the filters were not exposed to ambient air during transportation. During processing, filters were first dismantled to allow the filter membrane to be extracted. 1 ml of Bioline Lysis Buffer RLY (Bioline, Alexandria, Australia) was then added to the filter membrane in a tube, followed by centrifugation for 2 min at 2000 rpm. The full 1 mL of eluate was collected after the final spin and stored at − 20 °C until RNA extraction. Viral RNA in the eluate was extracted using the Isolate II RNA Mini Kit (Bioline, Alexandria, Australia) as per manufacturer’s instructions before conversion to cDNA.

### Virus detection in filters

The reverse transcription polymerase chain reaction (RT-PCR) (Bioline, Alexandria, Australia) was performed on the extracted filter membrane to detect rhinovirus, respiratory syncytial virus, influenza virus A and B, parainfluenza virus (type 1, 2 and 3) and human metapneumovirus. As an internal control, positive viral cDNA was included in each PCR assay. All primer sequences are provided in the (Additional file 1: Table S1).

Extracted RNA was converted to cDNA using the Bioline SensiFAST cDNA Synthesis Kit (Bioline, Alexandria, Australia) as per manufacturer’s instructions, with 8 μl of extracted RNA, 7 μl of DPEC water, 4 μl reaction buffer, 1 μl of reverse transcriptase added to each reaction to make a total volume of 20 μl. The PCR assay was performed as follows: all samples were run in triplicate, with 2 μl of cDNA template added to Bioline SensiFAST Probe Hi-ROX Master Mix. Specific primers and probes (Table 1) for each virus were added to the PCR assay along with DEPC water. The dual-labelled probes utilised the FAM fluorophore and BHQ-1 quencher. These samples were run on the StepOnePlus Real-Time PCR System (Applied Biosystems, California, USA) for 40 cycles. The threshold was automatically detected based on amplification. Positive viral samples and negative controls were run individually for each assay.

**Table 1:** Virus inoculation and subsequent recovery by PCR

Virus	Treatment	Conditions	1 week	2 weeks	4 weeks
Influenza virus	Negative controls^a^		No	No	No
	Virus added	20 °C	Detected	No	No
	Virus added	− 20 °C	Detected	Detected	Detected
Rhinovirus	Negative controls^a^		No	No	No
	Virus added	20 °C	Detected	No	No
	Virus added	− 20 °C	Detected	Detected	Detected

10 uL of viral stock was inoculated onto each ventilator filter. These filters were then stored at either room temperature (20 °C) or low temperature (− 20 °C) for 1, 2 or 4 weeks. Triplicates were stored for each condition

^a^Negative controls did not have any virus particles added to the filter

### Additional laboratory tests

In addition to ventilator filters, clinical respiratory samples were collected from each patient, including a nasopharyngeal swab, tracheal aspirate and/or bronchoalveolar fluid. Multiplex viral PCR (BioFire FilmArray, Salt Lake City, USA) was performed to detect the presence of rhinovirus, respiratory syncytial virus, influenza virus A and B, parainfluenza virus (type 1, 2 and 3) and human metapneumovirus in these samples. Assay characteristics and methodology of this multiplex viral PCR have been previously published [3–5]. This clinical testing was performed by the hospital laboratory scientists, separate from the researchers who performed the PCR assay on the ventilator filters. The researchers who performed the PCR assay on the ventilator filters were blind to the results of the multiplex viral PCR and vice versa. Tests for bacterial pathogens were also carried for each patient, including both typical and atypical respiratory pathogens.

### Serology and host response biomarker

To assess the host response to respiratory viruses, a blood sample was taken from each patient to measure (1) serological changes and (2) biomarker IFI27 changes. For the serological test, a positive seroconversion to influenza virus is defined as a low baseline antibody titre (< 1:10) followed by an increase (> 4 fold) in antibody titre between the two blood samples. For the *IFI27* biomarker, an increased *IFI27* gene expression in peripheral blood indicates an immune response to a specific respiratory virus with the following threshold cut-off values: influenza (> 74 fold change), parainfluenza virus (> 74 fold change), respiratory syncytial virus (> 40 fold change) and human metapneumovirus (> 40 fold change) [6].

### Statistical analysis

For continuous variables, comparisons between two groups were made using an unpaired two-tailed Student’s *t* test or the nonparametric Mann–Whitney *U* test, where appropriate. For categorical variables, comparisons between two groups were calculated using Fisher’s exact test. Statistical significance was defined as *p* < 0.05.

## Results

### Technical feasibility study

We first assessed the feasibility of detecting respiratory viruses in clean, unused ventilator filters. To this end, we inoculated two different respiratory viruses (influenza A and rhinovirus) using viral stock onto clean ventilator filters. These filters were then stored for 1, 2 and 4 weeks under different temperatures (room temperature or − 20 °C). After the storage period, we extracted the filter membrane from each filter casing and amplified viral nucleic acids using RT-PCR (as described in the Methods section). We detected viral nucleic acids after 1 week at room temperature and up to 4 weeks at − 20 °C (Table 1). Both influenza virus and rhinovirus were recovered in the inoculated filters (Table 1). This finding demonstrates the feasibility of using ventilator filters as a collection device, providing the basis for our sampling approach subsequently used in this study.

### Airborne viruses in ventilated patients

Having demonstrated the technical feasibility of our sampling method, we next investigated whether we could detect airborne viruses in mechanically ventilated patients. A total of 35 mechanically ventilated patients were recruited for the study. Full, detailed demographic and clinical features of the patients are provided in Table 2. In brief, 35 patients were admitted to the intensive care unit for the management of respiratory failure. Thirty of these 35 patients had pneumonia. Five patients had no evidence of infection—these patients acted as controls in the study. Infectious agents identified in the patients with pneumonia included viruses (*n* = 20), bacteria (*n* = 18), fungi (*n* = 1) and virus–bacteria co-infection (*n* = 8). A full list of identified infectious agents is provided in the (Additional file 2: Table S2). No infectious agents were identified in the control patients after a full microbiological and virology screen on each patient’s blood, urine and airway samples.

**Table 2:** Patient demographic and clinical characteristics

	Infected patients	Control patients
Number of patients	30	5
Age (years)^a^	58.6 (23–86)	52.6 (21–71)
Gender (male/female)	10/20	3/2
Infection types		
Bacterial	8	0
Viral	11	0
Bacteria–bacteria	2	0
Virus–bacteria	8	0
Virus–fungus	1	0
Severity and outcomes		
APACHE III scores^a^	67 (36–128)	57 (35–83)
Length of ventilation (days)^a^	8.7 (2–28)	3.8 (1–8)
Length of ICU stay (days)^a^	11.5 (2–37)	6 (2–11)
Length of hospital stay (days)^a^	16 (2–45)	8.6 (2–16)
Alive/dead	22/8	5/0

^a^Data are presented as mean and range (minimum–maximum)

A total of 70 ventilator filters were collected from the recruited patients, with one expiratory filter and one inspiratory filter collected from each patient. Airborne respiratory viruses were detected in nearly half of the filters (*n* = 33) using RT-PCR (Table 3). There were more viruses detected in the inspiratory filters (*n* = 18) than in the expiratory filters (*n* = 15). The most common virus detected was the influenza A virus (*n* = 29).

### Inspired air versus expired air

**Fig. 2:**
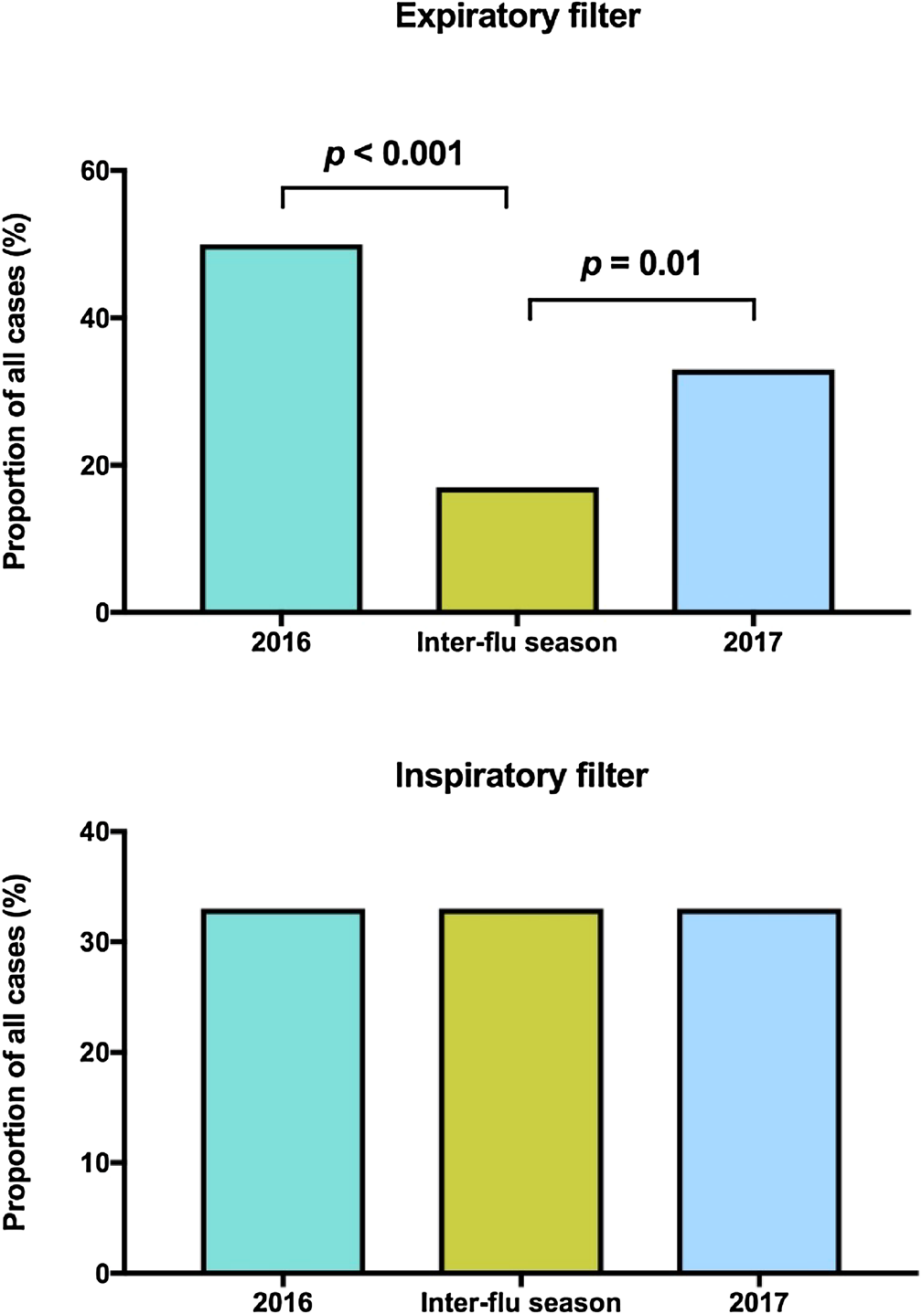
Seasonal changes in the frequency of detected viruses. The recruitment period covered two flu seasons in the Southern hemisphere one inter-seasonal period. “2016” refers to the first flu season (early July–late October 2016). “2017” refers to the second flu season (late July–mid-October 2017). “Inter-flu season” refers to the period in between the two seasons (November 2016 to early July 2017). *p* values were calculated using Fisher exact test. No difference was detected in the distribution of detected viruses in the inspiratory filters

We hypothesised that the expired air reflects the virus ecology inside the patients’ lungs. This means the distribution of viruses detected in the expiratory filters would resemble the viruses circulating in the local patient population, which would display seasonal fluctuations related to the onset/end of each flu season. To assess the impact of seasonal changes on virus detection in inspired/expired air, we divided the recruitment period into stages including (1) peak flu seasons and (2) off-peak flu season (Fig. 2). In this analysis, we found that the airborne viruses in the expiratory filters did show a seasonal pattern and matched the seasonal increase/decrease reported in our local institution (data not shown). In contrast, no seasonal fluctuation was observed in the influenza viruses detected in the inspiratory filters (Fig. 2), in keeping with the fact that inspired air came from the main hospital air supply (which is insulated from the viruses circulating in the local population) (Table 3).

**Table 3:** Airborne viruses in patients’ ventilator filters

	Virus	Inspiratory filters	Expiratory filters
Infected patients^a^	Influenza	14	9
	Rhinovirus	0	1
	Metapneumovirus	0	1
	Parainfluenza virus	1	1
Non-infected controls	Influenza	3	3
	Rhinovirus	0	0
	Metapneumovirus	0	0
	Parainfluenza virus	0	0
	Total	18	15

^a^Infected patients refer to pneumonia patients in whom a bacterium was identified by culture or a respiratory virus was identified either by PCR assay on respiratory secretions (e.g. nasopharyngeal swap, bronchoalveolar lavage fluid) or by serology on serum samples

### Sources of airborne viruses

Having demonstrated the presence of airborne viruses in mechanically ventilated patients, we next sought to identify the possible sources of these airborne viruses. A third of the cases (*n* = 9) were found in patients with an established diagnosis of respiratory virus infection, indicating that these patients were actively shedding viruses during the study period and some of these virus particles were detected by our method. In the remaining cases, the detected viruses were different from viruses identified in the respiratory secretions of the same patient, suggesting that these new viruses might come from either the ambient air (from routine change of the patient’s ventilator circuit) or from cross-contamination (staff or visitors).

### Host response to airborne viruses

**Fig. 3:**
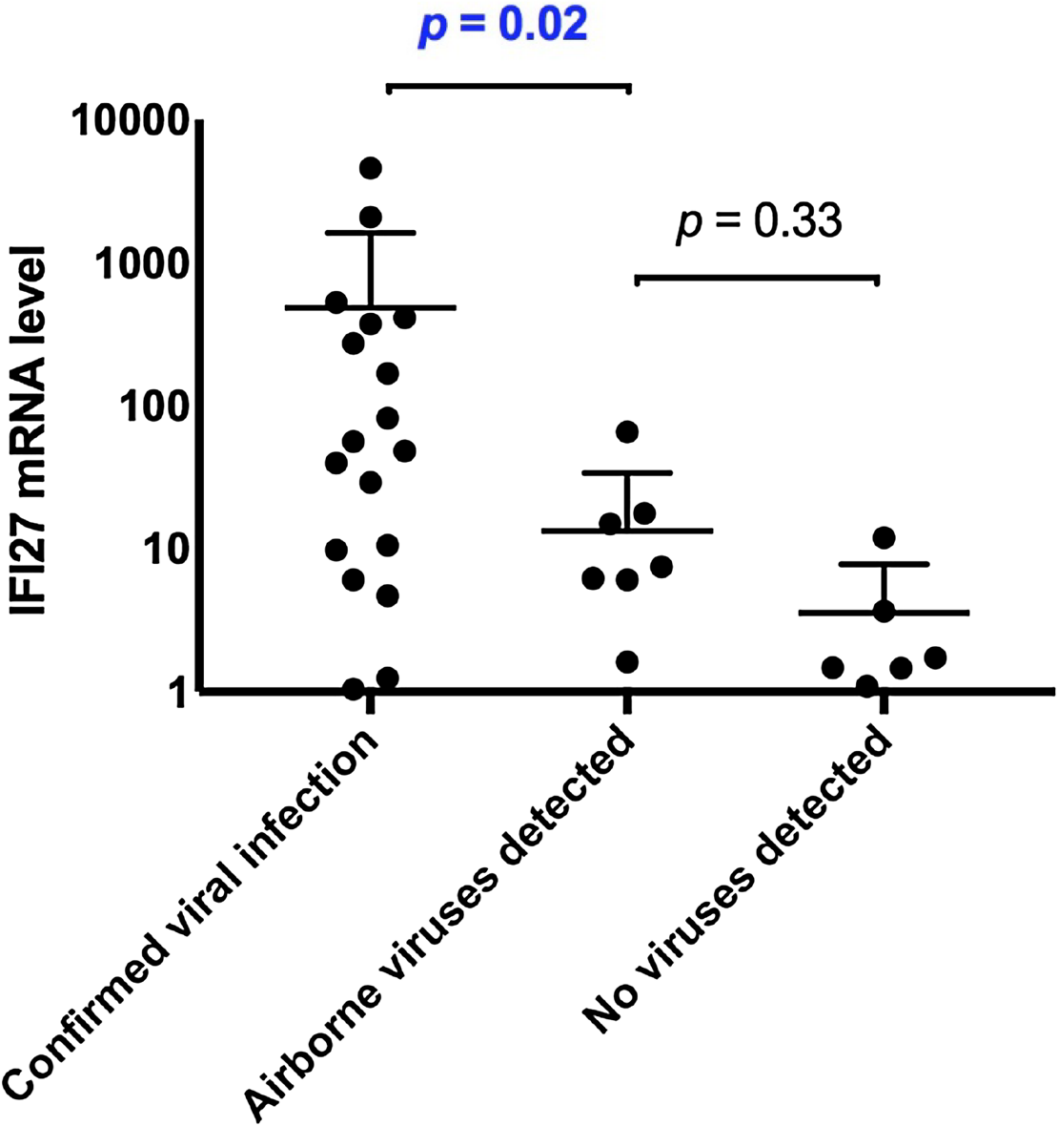
Host response biomarker and clinical outcomes. “*Confirmed viral infection*” group refers to all patients in whom a respiratory virus was identified in their respiratory secretions (e.g. nasopharyngeal swap, bronchoalveolar lavage fluid) or increased anti-viral titres in their serum as measured by serology. “*Airborne viruses detected*” group refers to patients in whom a new respiratory virus was detected in the inspiratory filter or the expiratory filter. “*No viruses detected*” group refers to patients in whom no respiratory virus was detected in the respiratory secretions, serum or the ventilator filters. The *IFI27 mRNA*-*expression* was measured by quantitative real-time PCR, and its level is expressed as fold change (relative to GAPDH). The *p* values were calculated using Kruskal–Wallis test (for comparison of multiple groups). The error bars are mean plus standard deviation

We next assessed the acute host response to the presence of airborne viruses in each patient. To this end, we analysed patients’ peripheral blood samples to measure the gene expression levels of the biomarker *IFI27*, an established marker of virus-induced immune response [3]. We compared the *IFI27* levels between patients with an established diagnosis of viral infection, patients in whom a new virus was detected (in their ventilator filters) and patients in whom no respiratory virus was found anywhere (in blood, respiratory secretions or ventilator filters). We found that *IFI27* levels were significantly elevated in those with confirmed respiratory viral infection (mean fold change = 483), confirming the presence of an immune response to the viral infection. In patients in whom a new virus was detected in their ventilator filters, the *IFI27* levels were low (mean fold change = 13) and not statistically different to patients who had no evidence of viral infection (Fig. 3); this result suggested an absence of virus-induced immune response in these patients (Table 4).

**Table 4:** Virus detected in each patient

Subjects	Status^a^	Airway	Serology	Inspiratory	Expiratory
1	Infected			Influenza	Influenza
2	Infected	RSV		Influenza	Influenza
3	Infected			Influenza	Influenza
4	Infected	Rhinovirus		Influenza	Influenza
5	Control				
6	Infected	Influenza		PIV	Influenza
7	Infected			Influenza	PIV
8	Infected	Rhinovirus			
9	Control			Influenza	Influenza
10	Infected				
11	Infected	Rhinovirus		Influenza	
12	Control			Influenza	
13	Infected	PIV			
14	Control			Influenza	Influenza
15	Infected	Rhinovirus		Influenza	Influenza
16	Infected	RSV		Influenza	
17	Infected				
18	Infected				
19	Infected				
20	Infected				
21	Infected	Influenza		Influenza	
22	Infected	Influenza			
23	Infected		Influenza		
24	Infected	Influenza			
25	Infected	HMPV			HMPV
26	Infected	Influenza			
27	Infected	Influenza		Influenza	
28	Infected	Influenza		Influenza	Influenza
29	Infected	Influenza			Influenza
30	Infected	Influenza	Influenza		Influenza
31	Control				Influenza
32	Infected	Influenza		Influenza	
33	Infected			Influenza	
34	Infected	Influenza		Influenza	Rhinovirus
35	Infected	Influenza	Influenza		

*RSV* respiratory syncytial virus, *HMPV* human metapneumovirus, *PIV* parainfluenza virus

^a^In this column, “Infected” refers to any of the following status; (1) bacterial infection, (2) viral infection or (3) viral–bacterial co-infection

## Discussion

This is the first report to assess the feasibility of using a novel sampling method to detect airborne respiratory viruses in a critically ill patient population. The results show that airborne viruses were present in 44% of the ventilator filters collected from mechanically ventilated patients. The vast majority of the detected airborne viruses (88%) were influenza viruses. In some cases, the airborne viruses detected reflected the carrier status of the patients, with the same virus found in both the ventilator filter and patients’ respiratory secretions. In other cases, where a new virus was detected, the clinical significance of these viruses remains unclear, since the affected patients showed no evidence of a virus-induced immune response.

A large number of studies have demonstrated that respiratory viruses (e.g. influenza viruses) are always present in the ambient air [2]. The importance of detecting airborne viruses present in the hospital environment is increasingly being recognised. Several recent studies have provided a direct demonstration that influenza viruses were present in aerosolised droplets from the tidal breathing of infected persons and in the air of the emergency department [7, 8]. During peak flu season, the concentration of airborne viruses in the environment rises to 5800–37,000 virus particles per m^3^. At this concentration, breathing air for 1 h is sufficient to cause clinical infection in a previously unexposed person [9]. Thus, monitoring airborne virus concentrations may be important in a high-risk clinical environment such as the intensive care unit, where many patients have immune-compromised status and an increased susceptibility to opportunistic infection. Furthermore, there is significant risk of droplet transmission if visitors or healthcare staff are infected with respiratory viruses and are in close contact with these critically ill patients. The method outlined in this study may provide a tool to monitor both airborne and droplet sized viral particles that mechanically ventilated patients are exposed to.

Monitoring airborne viruses requires a different approach than the conventional testing method of respiratory viruses. This difference is due to the fact that the concentration of viruses in the ambient air is much lower than that of the respiratory secretions (e.g. nasopharyngeal swab or bronchoalveolar lavage). Unfortunately, there is a lack of data on detection method specifically developed for airborne viruses. This study represents the first step towards developing a reliable, easy-to-perform method for airborne virus detection. Future studies should investigate whether such methods could increase the diagnostic yield of detecting viral aetiology in patients with community-acquired pneumonia or whether such methods could prevent hospital-acquired viral infection in mechanically ventilated patients.

There are additional challenges with investigating airborne respiratory viruses in critically ill patients due to experimental difficulties in sampling aerosolised virus particles, including the potential inactivation of viruses by current sampling methodology [10]. To overcome this sampling difficulty, we used ventilator filters as a collection device, since these filters collect a large volume of air which potentially increases the yield of detected virus particles. This approach was first tested in the pilot phase of this study, in which we inoculated live viruses into clean, unused filters. Days later, we were able to recover the same viruses in the filters. This finding provides strong support for using this approach in our study. The result is also in keeping with findings from a previous study by Heuer et al., who demonstrated that aerosolised influenza virus particles could be trapped inside ventilator filters [11]. In Heuer’s study, three different brands of commercially available filters were tested; all showed that the filters could successfully collect airborne viruses. Whilst the Heuer study was designed as an in vitro study, our study provides real-world data in a clinical setting. Collectively, both the in vitro and the clinical data confirm the technical feasibility of using ventilator filters as a collection device.

We used a RT-PCR assay to detect viruses in the present study, which is the method of choice for airborne viruses suggested by the established literature [1]. We purposefully adopted a more sensitive detection threshold (cycle threshold (Ct) value of 37–38) in order to quantify the lowest background virus level inside the ventilator circuits. This information allowed us assess the baseline risk level of airborne viruses in our clinical environment. It is important to note that the low detection threshold used in this study detects viruses at concentrations less than 500 virus particles per m^3^ [12]. Such a low virus concentration is generally insufficient to breach the normal defence barrier of the host’s airways and, hence, is unlikely to cause clinical infection in the affected individual. This helps explain the observation that there was no evidence of virus-induced immune response in patients in whom a virus was detected in the filters and the clinical course/outcomes of these patients did not differ from the control patients.

The current study has some limitations, first of which was a small sample size and selected cohort of patients, meaning the generalisability of the findings to other patient populations is limited. As part of this, it was impossible to determine the original size of the viral particles that were collected on ventilator filters making it difficult to delineate whether this was detection of airborne transmission, droplet transmission or a combination of the two. Secondly, we did not quantify the number of virus particles in the collected samples (ventilator filter or respiratory samples). As a result, no information was available regarding the precise viral load in each sample. Thirdly, we did not perform sequencing of the identified viruses, making it difficult to know with certainty the exact source of each virus. Fourthly, we did not assess whether the detected virus particles could replicate in human cells and therefore had no information regarding the viruses’ viability or infectivity, both of which are clinically important. A further limitation of this study is that 24 h was used as a sampling time; it is possible that a longer sampling time (e.g. 48 h, 72 h) may increase the yield of virus detection.

## Conclusion

This preliminary study shows that it is technically feasible to detect airborne viruses in the ventilator filters collected from patients receiving mechanical ventilation. Our findings provide important baseline data regarding the presence of airborne viruses in critically ill patients and may help inform the design of future studies in a similar setting.

## Additional files

**Additional file 1: Table S1.** Virus-specific primer and probe sequences for real-time PCR.

**Additional file 2: Table S2.** Pathogens identified in patient samples^ψ^ (excluding ventilator filers samples).

## Abbreviations

Ct: cycle threshold
RT-PCR: reverse transcriptase polymerase chain reaction

## References

[1] Nikitin N, Petrova E, Trifonova E, Karpova O (2014). Influenza virus aerosols in the air and their infectiousness. Adv Virol.

[2] Hall CB (2007). The spread of influenza and other respiratory viruses: complexities and conjectures. Clin Infect Dis.

[3] Chen H, Weng H, Lin M, He P, Li Y, Xie Q, Ke C, Jiao X (2017). The clinical significance of FilmArray respiratory panel in diagnosing community-acquired pneumonia. Biomed Res Int.

[4] Rogers BB, Shankar P, Jerris RC, Kotzbauer D, Anderson EJ, Watson JR, O’Brien LA, Uwindatwa F, McNamara K, Bost JE (2015). Impact of a rapid respiratory panel test on patient outcomes. Arch Pathol Lab Med.

[5] Andrews D, Chetty Y, Cooper BS, Virk M, Glass SK, Letters A, Kelly PA, Sudhanva M, Jeyaratnam D (2017). Multiplex PCR point of care testing versus routine, laboratory-based testing in the treatment of adults with respiratory tract infections: a quasi-randomised study assessing impact on length of stay and antimicrobial use. BMC Infect Dis.

[6] Tang BM, Shojaei M, Parnell GP, Huang S, Nalos M, Teoh S (2017). A novel immune biomarker IFI27 discriminates between influenza and bacteria in patients with suspected respiratory infection. Eur Respir J.

[7] Tellier R (2009). Aerosol transmission of influenza A virus: a review of new studies. J R Soc Interface.

[8] Stelzer-Braid S, Oliver BG, Blazey AJ, Argent E, Newsome TP, Rawlinson WD (2009). Exhalation of respiratory viruses by breathing, coughing, and talking. J Med Virol.

[9] Yang W, Elankumaran S, Marr LC (2011). Concentrations and size distributions of airborne influenza A viruses measured indoors at a health centre, a day-care centre and on aeroplanes. J R Soc Interface.

[10] Verreault D, Moineau S, Duchaine C (2008). Methods for Sampling of Airborne Viruses. Microbiol Mol Biol Rev.

[11] Heuer JF, Crozier TA, Howard G, Quintel M (2013). Can breathing circuit filters help prevent the spread of influenza A (H1N1) virus from intubated patients?. GMS Hyg Infect Control..

[12] Stone B, Burrows J, Schepetiuk S, Higgins G, Hampson A, Shaw R (2004). Rapid detection and simultaneous subtype differentiation of influenza A viruses by real time PCR. J Virol Methods.

