# Supplementary figures and images for: A novel sampling method to detect airborne influenza and other respiratory viruses in mechanically ventilated patients: a feasibility study
Source: Ann Intensive Care. 2018 Apr 17;8:45. doi: 10.1186/s13613-018-0396-4 (PMC5904094; doi:10.1186/s13613-018-0396-4)

**Additional file 1: Table S1. Virus-specific primer and probe sequences for real time PCR.**


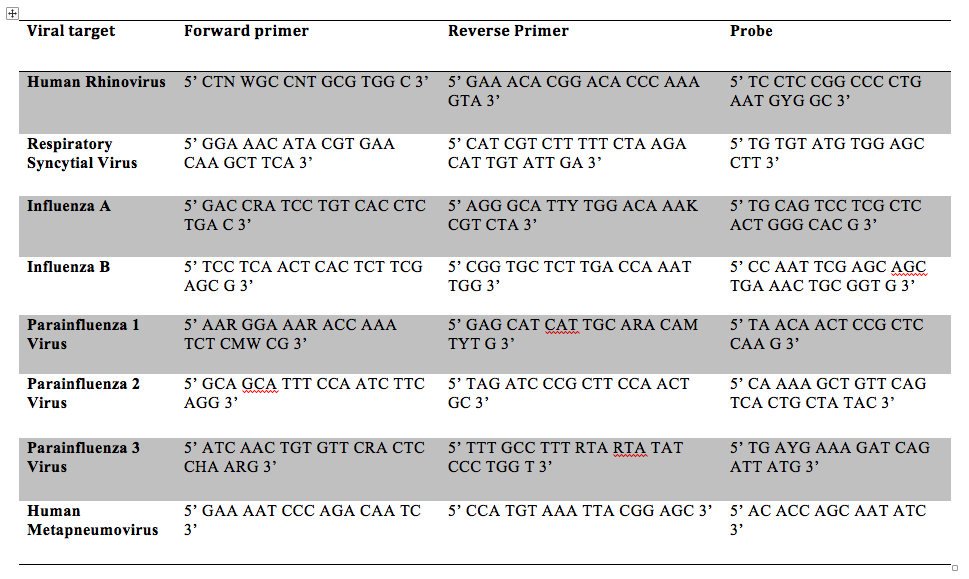

Supplement: Supplementary file 1 — Additional file 1: Table S1. Virus-specific primer and probe sequences for real-time PCR. [file 13613_2018_396_MOESM1_ESM.docx]
