# Supplementary material for: A novel sampling method to detect airborne influenza and other respiratory viruses in mechanically ventilated patients: a feasibility study
Source: Ann Intensive Care. 2018 Apr 17;8:45. doi: 10.1186/s13613-018-0396-4 (PMC5904094; doi:10.1186/s13613-018-0396-4)
Supplement: Supplementary file 2 — Additional file 2: Table S2. Pathogens identified in patient samplesψ (excluding ventilator filers samples). [file 13613_2018_396_MOESM2_ESM.docx]

| Additional file 2: Table S2. Pathogens identified in patient samples ^ψ^  (excluding ventilator filers samples) | |
| --- | --- |
| Organisms | **Number** |
|  |  |
| Influenza A | 6 |
| *Strep. pneumoniae* | 2 |
| *Chlamydia/Coxiella* | 1 |
| E. coli | 1 |
| *Haemophilus influenzae/Staph. aureus* | 1 |
| Human Metapneumovirus | 1 |
| Influenza A/*Klebsiella* | 1 |
| Influenza A/*Pseudomonas aeruginosa* | 1 |
| Influenza A/*Staph. aureus* | 1 |
| Influenza B | 1 |
| Influenza B/*Staph. aureus* | 1 |
| Influenza B/*Strep. pneumoniae* | 1 |
| *Mycoplasma pneumoniae* | 1 |
| Parainfluenza virus | 1 |
| *Pneumocystis jirovecii*/Rhinovirus | 1 |
| *Pseudomonas aeruginosa* | 1 |
| Rhinovirus | 1 |
| Respiratory syncytial virus | 1 |
| Respiratory syncytial virus/*Staph. aureus* | 1 |
| *Strep. pneumoniae*/*Straph.* aureus/Rhinovirus | 1 |
| *Strep. pneumoniae*/Rhinovirus | 1 |
| Unidentified bacteria | 3 |
|  |  |

**^ψ^** Patient samples included nasopharyngeal swab, sputum, tracheal aspirate or bronchoalveolar lavage fluid.
